# Relationship between bone mineral density and ovarian function and thyroid function in perimenopausal women with endometriosis: a prospective study

**DOI:** 10.1186/s12905-022-01711-3

**Published:** 2022-04-27

**Authors:** Mari Uehara, Osamu Wada-Hiraike, Mana Hirano, Kaori Koga, Noriko Yoshimura, Sakae Tanaka, Yutaka Osuga

**Affiliations:** 1 Department of Obstetrics and Gynecology, The University of Tokyo, Bunkyo-ku, Tokyo, 113-8655 Japan; 2 Department of Preventive Medicine for Locomotive Organ Disorders, 22nd Century Medical and Research Center, The University of Tokyo, Bunkyo-ku, Tokyo, 113-8655 Japan; 3 Department of Sensory and Motor System Medicine, The University of Tokyo, 7-3-1 Hongo, Bunkyo-ku, Tokyo, 113-8655 Japan

**Keywords:** Ovarian reserve, Endometriosis, Perimenopause, Osteoporosis, Bone mineral density

## Abstract

**Background:**

In women with endometriosis, the association between ovarian function, hormones, and bone mineral density (BMD) is unclear. Therefore, this study aimed to elucidate the association between changes in bone mineral density (BMD) and clinical data, such as ovarian reserves, in perimenopausal women with endometriosis.

**Methods:**

In this prospective study, we evaluated 207 female patients who visited the Department of Obstetrics and Gynecology at the University of Tokyo Hospital between December 2015 and December 2020. We included patients aged ≥ 40 years with a history of endometriosis or who presented with endometriosis lesions. Patients with a history of smoking, steroid administration, autoimmune diseases, dyslipidaemia, and heart disease were excluded. During the study period, patients who underwent two tests, an initial and a follow-up test (n = 142, average age: 45.02 years, average BMD: 1.16 g/cm^2^), were evaluated at regular intervals based on the annual rate of change in BMD.

**Results:**

There was a weak negative correlation between the follicle-stimulating hormone (FSH) and BMD and a weak positive correlation between the anti-Müllerian hormone (AMH) and BMD. The annual rate of change in BMD showed a very weak correlation with thyroid-stimulating hormone (TSH) levels. A large decline in BMD was associated with high TSH levels and higher average age at menopause. Patients with higher TSH exhibited a higher rate of decrease in BMD than those without.

**Conclusions:**

High FSH or low AMH levels are associated with decreased BMD. Decreased ovarian reserve is associated with decreased BMD in perimenopausal women with endometriosis. High TSH levels increase the risk of BMD loss. This finding may suggest that women with endometriosis should undergo bone scanning to rule out the possibility of reduced bone mass and subsequent increased risk of fracture.

## Background

One of the aetiologies of osteoporosis is a decrease in oestrogen levels due to a decrease in ovarian function [1–3]. In addition to suppressing bone resorption by acting directly on osteoclasts, oestrogen suppresses osteoclast differentiation and bone resorption by inhibiting the expression of osteoclast differentiation factors [4]. The decrease in bone strength and bone quality, defined by bone mineral density (BMD), progresses with age. After menopause, when patients enter a low oestrogen state, bone resorption increases, and BMD decreases, making women more prone to osteoporosis at this stage [1, 5, 6].

Endometriosis is a chronic inflammatory disease characterised by the presence of endometrial-like tissue outside the uterus [7]. It is believed to affect 10% of women of reproductive age and form lesions in areas, such as the ovaries and peritoneum, causing dysmenorrhoea, chronic pelvic pain, dyspareunia, and infertility [7–9]. Endometriosis is an oestrogen-dependent disease because oestrogen plays an important role in its pathophysiology. Endometriosis enhances oestrogen receptor expression and progesterone resistance in endometrial tissues [10]. Oestrogen promotes the transplantation of endometrial tissue into the peritoneum, thereby affecting proliferation and immortalisation, and also causes local and systematic inflammation [8, 11]. Of the inflammatory factors, it has been reported that autoimmunity plays a major role in the development of endometriosis, and the relationship between thyroid autoimmunity and endometriosis has been highlighted [12, 13].

In addition to oestrogen, follicle-stimulating hormone (FSH), luteinising hormone (LH), which are factors that depend on the menstrual cycle, and anti-Müllerian hormone (AMH) have been attracting considerable attention in recent years for assessing ovarian function. AMH is produced by the granulosa cells of follicles, can be measured in the serum, and is independent of the menstrual cycle. Ovarian reserve refers to ovarian function characterised by the quantity and quality of follicles; AMH has been shown to be an indicator of ovarian reserve, which is useful to optimise ovarian stimulation in fertility treatment, preserve fertility in young cancer patients, and predict the timing of menopause onset [14–17].

In addition to symptomatic treatment to suppress pain, pharmacotherapy for endometriosis includes hormone therapy to suppress oestrogen levels [9]. However, long-term hormone therapy can lead to a decrease in BMD. In addition, it has been demonstrated that surgical treatment may reduce ovarian function not only with radical oophorectomy but even when only the ovarian lesions are removed. Therefore, in patients with endometriosis, changes in ovarian function may affect bone metabolism and cause changes in BMD.

Previous studies have revealed that women with endometriosis did not exhibit a decrease in BMD compared with women of the same age without endometriosis [18, 19] and that long-term fracture risks did not increase in women with endometriosis [20]. Meanwhile, regarding the decrease in BMD as a side effect of hormone therapy, the impact of add-back therapy using gonadotropin-release hormone analogues and oestrogen preparations for bone protection [21], as well as the decrease in BMD due to long-term administration of Dienogest, a progestin preparation [22] have been investigated. Another report suggested that BMD was higher after ovariectomy for deep endometriosis than after ovariectomy for other indications [23]. However, almost no studies have investigated the relationship between ovarian function and BMD changes in women with endometriosis. Elucidating the association between BMD loss and ovarian function in women with endometriosis could be beneficial for preventing BMD loss and subsequent osteoporosis.

Therefore, the purpose of this study was to clarify the effects of ovarian function and endocrinological factors related to ovarian function on BMD reduction in perimenopausal patients with endometriosis. We investigated the association of BMD with the levels of FSH, AMH, and thyroid hormone in perimenopausal patients with endometriosis.

## Methods

### Study design and participants

This prospective cohort study included 207 patients who visited the Department of Obstetrics and Gynecology of the University of Tokyo Hospital between December 2015 and December 2020. Following the approval of the study by the University of Tokyo Research Ethics Committee, we obtained written informed consent from all participants. To clarify the effects of ovarian function and endocrinological factors related to ovarian function on BMD reduction in perimenopausal patients with endometriosis, we included patients aged ≥ 40 years who had a history of endometriosis or presented with endometriosis lesions at the time of study participation. Patients with a history of endocrine disorders, such as diabetes, smoking, and steroid administration, factors which could affect bone metabolism, were excluded.

### Physical assessment and laboratory analysis

Patient height and body weight were measured as physical measurements to calculate the body mass index (BMI). An ankle-brachial pressure index/pulse wave test was performed, and the upper arm’s systolic and diastolic blood pressures were measured. We analysed blood samples from participants using the chemiluminescence enzyme immunoassay method to measure FSH and oestradiol. The electrochemiluminescence immunoassay method was used to measure free T_4_ (thyroxine), thyroid-stimulating hormone (TSH), and AMH as ovarian function markers. The enzyme immunoassay method was used to measure tartrate-resistant acid phosphatase-5b (TRACP-5b) as a marker of bone resorption. If the test result was below the measurement limit, the result was corrected to the lower limit before the analysis. BMD was measured at the lumbar spine (L2-L4) using the dual-energy X-ray absorptiometry (DXA) method (Discovery DXA System, Hologic, Inc., Marlborough, MA). Using the medical records, we collected information about patient age at testing, history of hormone therapy, surgical history, number of remaining ovaries, whether the patient had undergone menopause, and if so, the age at which menopause occurred.

During the study period, 142 patients (68.6%) who underwent two tests, an initial test and a follow-up test, were analysed at regular intervals based on the annual rate of BMD change. Of the 65 deviating patients, 19 patients ended their outpatient visits to our hospital because of relocation, and 46 patients refused follow-up testing. These patients were excluded from the analysis. We calculated the period between the initial and follow-up testing (years) and used the following formula from previous literature to determine the annual rate of BMD change [24].

$${\text{Annual Rate of BMD change}}\;{\text{(\% /year) = }}\frac{(BMD\;of\;second\;measurement) - (BMD\;of\;Initial\;measurement)}{{(BMD\;of\;Initial\;measurement) \times (year)}} \times 100$$

The patients were divided according to tertiles based on the annual rate of BMD change into severe bone loss, moderate bone loss, and mild bone loss groups. We compared the physical measurements at initial testing, haematological results, history of hormone therapy, surgical history, number of remaining ovaries, and age at menopause among the groups.

All statistical analyses were performed using the STATA statistical package (Stata Corporation, College Station, TX). Statistical comparisons between groups were performed using paired t-test and analysis of variance. A multiple comparison test was performed. Multinomial logistic analysis adjusted for age and body weight was conducted using the mild bone loss group as a reference. Statistical significance was set at *p* < 0.05.

## Results

### Baseline characteristics

Table 1 shows the characteristics of the study participants. The average age was 45.02 years, and the average BMD was 1.16 g/cm^2^. The remaining ovaries were on both sides in 129 patients, one side in 73 patients, and none (both sides removed) in 5 patients. There were 70 (33.82%) postmenopausal patients, and the average age at menopause was 46.84 years. In addition, there were 106 patients with a history of hormone therapy within the previous year, and a total of 171 patients had a history of ovarian surgery.

**Table 1:** Baseline characteristics of participants

	N	Mean
Age (years)	207	45.02 ± 2.73
Weight (kg)	207	55.31 ± 9.02
Height (cm)	207	159.23 ± 5.67
Body mass index (kg/m^2^)	207	21.80 ± 3.27
Systolic blood pressure (mmHg)	198	121.98 ± 13.04
Diastolic blood pressure (mmHg)	198	78.98 ± 10.29
FSH (mIU/mL)	202	33.42 ± 41.84
E_2_ (pg/mL)	201	92.98 ± 107.48
TSH (μIU/mL)	198	1.72 ± 1.11
Free T_4_ (μg/dL)	199	1.09 ± 0.17
AMH (ng/mL)	166	0.36 ± 0.57
TRACP-5b (mU/dL)	174	242.42 ± 111.96
BMD (g/cm^2^)	207	1.16 ± 0.15
Current hormonal therapy (%)	106	51.21
Surgical treatment (%)	171	82.61
*Residual ovary*		
Bilateral (%)	129	62.32
Unilateral (%)	73	35.27
None (%)	5	2.42
Postmenopausal (%)	70	33.82
Age at menopause (years)		46.84 ± 3.33

Data were presented as means ± standard deviation or number of cases (%)

Current hormonal therapy is defined as the administration of hormonal agents within 1 year before the initial measurement

BMD was measured at the lumbar spine using dual-energy X-ray absorptiometry (DXA)

BMD, bone mineral density; FSH, follicle-stimulating hormone; AMH, anti-Müllerian hormone; TSH, thyroid-stimulating hormone; E_2_, oestradiol; T_4_, thyroxine; TRACP-5b, tartrate-resistant acid phosphatase-5b

First, we studied factors affecting BMD. Table 2a shows the correlation between BMD and other factors at the initial testing. Pearson correlation analysis identified a significant correlation between BMD and the following factors: age exhibited a very weak negative correlation (r =  − 0.1523, *p* = 0.0285), body weight, and BMI showed a positive correlation (r = 0.4539; *p* < 0.0001 and r = 0.3996; *p* < 0.0001, respectively), whereas FSH showed a weak negative correlation (r =  − 0.3126, *p* < 0.0001) with BMD. Similarly, free T_4_ showed a very weak negative correlation (r =  − 0.1604, *p* = 0.0236) and AMH showed a weak positive correlation (r = 0.2183, *p* = 0.047) with BMD. FSH and AMH showed a significant association with BMD according to the multiple regression analysis adjusted for age and body weight (FSH: β =  − 0.00071, *p* = 0.0038; AMH: *p* = 0.018) (Table 2b).

**Table 2:** (a) Correlation between BMD and initial measurement. (b) Correlations between BMD, FSH, and AMH adjusted for age and weight: partial regression coefficients

(a)		
	R^2^	*P*-value
Age (years)	0.023	0.0285
Weight (kg)	0.206	< 0.0001
Height (cm)	0.194	0.0052
Body mass index	0.160	< 0.0001
Systolic blood pressure (mmHg)	0.017	0.0647
Diastolic blood pressure (mmHg)	0.019	0.0559
FSH (mIU/mL)	0.098	< 0.0001
E_2_ (pg/mL)	0.004	0.371
TSH (μIU/mL)	0.004	0.3604
Free T_4_ (μg/dL)	0.026	0.0236
AMH (ng/mL)	0.048	0.0047
TRACP-5b (mU/dL)	0.072	0.0003

(b)			
	N	Β	*P*-value
FSH (mIU/mL)	202	− 0.00071	0.0038
AMH (ng/mL)	166	0.05006	0.018

AMH, anti-Müllerian hormone; BMD, bone mineral density; E_2_, oestradiol; FSH, follicle-stimulating hormone; T_4_, thyroxine; TSH, thyroid-stimulating hormone; TRACP-5b, tartrate-resistant acid phosphatase-5b

### Analysis of the changes in BMD

Next, we analysed the rate of change in BMD. Table 3 summarises the data of 142 patients analysed to determine the annual rate of BMD change. The average BMD of initial and follow-up testing ware 1.15 ± 0.16 g/cm^2^ and 1.14 ± 0.15 g/cm^2^, respectively. The BMD between the initial and follow-up measurements BMD is significantly different (*p* = 0.023). The average period between the initial and follow-up testing was 1.41 ± 0.53 (range: 0.25–2.75) years. The changes in BMD and the annual rate of BMD change were 0.02 g/cm^2^ and − 0.53%/year, respectively.

**Table 3:** Physical and hormonal characteristics of initial and follow-up measurements for participants who underwent follow-up measurements

	Initial measurement		Follow-up measurement	
	N	Mean	N	Mean
Age (years)	142	45.01 ± 2.63	142	46.42 ± 2.75
Weight (kg)	142	55.60 ± 9.57	142	55.9 ± 9.82
Height (cm)	141	159.05 ± 5.79	142	158.89 ± 5.85
Body mass index	141	21.92 ± 3.37	142	22.12 ± 3.51
Systolic blood pressure (mmHg)	138	122.26 ± 11.65	140	121.54 ± 12.48
Diastolic blood pressure (mmHg)	137	78.83 ± 9.40	140	78.46 ± 9.51
FSH (mIU/mL)	139	32.74 ± 41.58	136	42.37 ± 40.48
E_2_ (pg/mL)	138	91.51 ± 108.28	136	78.08 ± 99.07
TSH (μIU/mL)	136	1.79 ± 1.17	131	1.76 ± 0.94
Free T_4_ (μg/dL)	136	1.08 ± 0.16	131	1.15 ± 0.17
AMH (ng/mL)	117	0.35 ± 0.59	93	0.18 ± 0.40
TRACP-5b (mU/dL)	121	244.67 ± 117.58	99	217.63 ± 117.75
BMD (g/cm^2^)	142	1.15 ± 0.16	141	1.14 ± 0.15
BMD change (g/cm^2^)	141	0.02 ± 0.02		
BMD rate of change (%/year)	141	− 0.53 ± 2.55		
Interval between initial and follow-up visit (years)	141	1.41 ± 0.53		
Current hormonal therapy (%)	83	58.45		
Surgical treatment (%)	122	85.92		
*Residual ovary*				
Bilateral (%)	90	63.38		
Unilateral (%)	48	33.80		
None (%)	4	2.82		
Postmenopausal (%)	53	37.32		
Age at menopause (years)		46.77 ± 3.41		

Data are presented as means ± SD or number of cases (%)

AMH, anti-Müllerian hormone; BMD, bone mineral density; E_2_, oestradiol; FSH, follicle-stimulating hormone; T_4_, thyroxine; TSH, thyroid-stimulating hormone; TRACP-5b, tartrate-resistant acid phosphatase-5b

We examined factors that affected the rate of BMD change. Table 4 shows the correlation between changes in BMD and physical/endocrinological factors measured at the initial test. TSH levels showed a very weak negative correlation (*p* = 0.0221) with BMD. Other factors were not significantly correlated with the rate of change in BMD.

**Table 4:** Correlation between change in annual rates of BMD (%/year) and initial measurement

	R^2^	*P*-value
Age (years)	0.01925	0.1009
Weight (kg)	0.001046	0.7034
Height (cm)	0.01331	0.1747
Body mass index	0.0002558	0.8512
Systolic blood pressure (mmHg)	0.006861	0.3359
Diastolic blood pressure (mmHg)	0.0004704	0.8021
FSH (mIU/mL)	0.002262	0.5796
E_2_ (pg/mL)	0.003724	0.4771
TSH (μIU/mL)	0.03873	0.0221
Free T_4_ (μg/dL)	0.0007232	0.7569
AMH (ng/mL)	0.003598	0.5224
TRACP-5b (mU/dL)	0.0001008	0.913
BMD (g/cm^2^)	0.02527	0.0597

AMH, anti-Müllerian hormone; BMD, bone mineral density; E_2_, oestradiol; FSH, follicle-stimulating hormone; T_4_, thyroxine; TSH, thyroid-stimulating hormone; TRACP-5b, tartrate-resistant acid phosphatase-5b

### Multinomial logistic regression analysis: comparison of the rate of change in BMD by tertiles

**Fig. 1:**
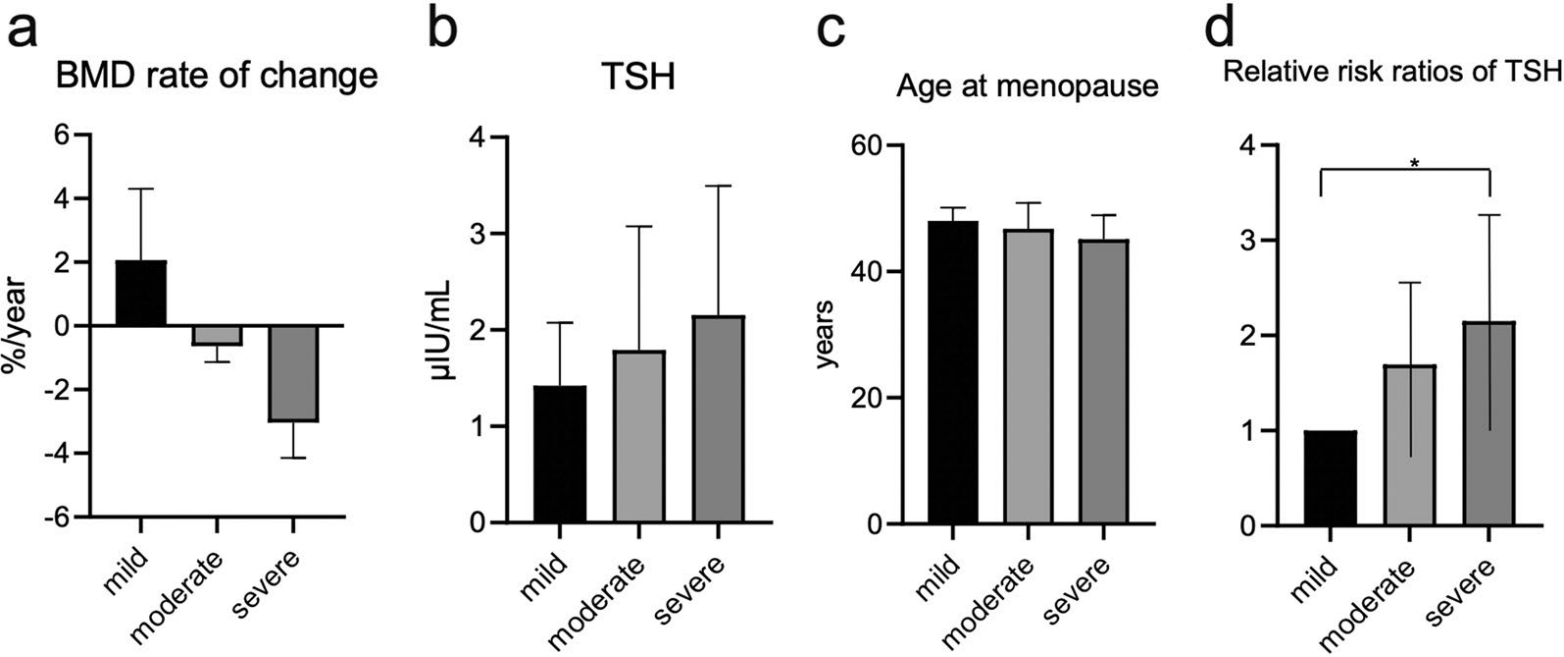
Comparison of the annual rates of change in the BMD, TSH and age at menopause. **a** BMD rates of change among the mild, moderate and severe groups. There were significant differences among the groups. **b** TSH levels in the three groups. There were significant differences among the groups. **c** Age at menopause in the three groups. There were significant differences among the groups. **d** Comparison of the relative risk ratios of TSH categorised by BMD rate of change. Values were adjusted for age and body weight. Error bars represent 95% confidence interval for the mean changes. Mild, mild bone density loss group; moderate, moderate bone density loss group; severe, severe bone density loss group; BMD, bone mineral density; TSH, thyroid-stimulating hormone. *p < 0.05

We performed a multinomial logistic regression analysis to elucidate the risk factors for annual BMD decrease. The physical/endocrinological factors of each group of participants divided according to tertiles based on the rate of BMD change as per initial and follow-up testing are presented in Table 5. The average ages of the mild bone loss, moderate bone loss, and severe bone loss groups were 44.68 years, 45.09 years, and 45.34 years, respectively, with no significant difference between them. Similarly, the average body weights were 54.33 kg, 56.60 kg, and 55.75 kg, respectively, for the three groups, exhibiting no significant differences. The average BMD were 1.11 ± 0.19 g/cm^2^, 1.17 ± 0.14 g/cm^2^, and 1.15 ± 0.13 g/cm^2^, respectively, for the three groups, exhibiting no significant differences (in mild vs moderate bone loss group, *p* = 0.21, 95% confidence interval [CI] − 0.032 to 0.16, in moderate vs severe bone loss group, *p* = 0.86, 95% CI − 0.094 to 0.055, and in mild vs severe bone loss group, *p* = 0.50, 95% CI − 0.048 to 0.13). Figure 1a depicts the annual rate of BMD change in each group. The annual rates of BMD change in the mild, moderate, and severe bone loss groups were 2.07 ± 2.24%/year, 0.64 ± 0.50%/year, and 3.03 ± 1.12%/year, respectively. Figure 1b shows the TSH values for each group. The TSH values of the three groups were 1.42 ± 0.65 μIU/mL, 1.79 ± 1.28 μIU/mL, and 2.16 ± 1.34 μIU/mL, respectively. There were significant differences between the groups. A greater rate of decrease in BMD was associated with a higher TSH level. Similarly, Fig. 1c shows the average age at menopause in each group. The average age at menopause was 48.04 years, 46.80 years, and 45.13 years, respectively, and exhibited a significant difference. A greater rate of decrease in BMD was associated with a significantly higher average age at menopause. Figure 1d illustrates the results of the multinomial logistic regression analysis of TSH levels corrected for age and BMI. The relative risk of the two remaining groups in comparison with the mild bone loss group was 1.69 ± 0.45 (95% confidence CI 1.01–2.85) in the moderate bone loss group and 2.15 ± 0.57 (95% CI 1.28–3.61) in the severe bone loss group, indicating a significant difference. Multinomial logistic regression analysis with other factors, including FSH and AMH, did not identify a significant increase in the risk of bone loss.

**Table 5:** Physical and hormonal characteristics among women categorised based on the annual rates of change in BMD

	Mild bone density loss (n = 47)	Moderate bone density loss (n = 47)	Severe bone density loss (n = 47)
Age (years)	44.68 ± 2.45	45.09 ± 2.53	45.34 ± 2.88
Weight (kg)	54.33 ± 9.47	56.60 ± 9.46	55.75 ± 9.90
Height (cm)	158.29 ± 5.78	159.69 ± 5.65	159.38 ± 5.91
Body mass index	21.66 ± 3.48	22.10 ± 3.26	21.91 ± 3.38
Systolic blood pressure (mmHg)	121.72 ± 10.69	119.91 ± 11.63	124.50 ± 11.77
Diastolic blood pressure (mmHg)	79.30 ± 9.82	77.36 ± 9.88	79.39 ± 8.26
FSH (mIU/mL)	37.34 ± 50.03	21.60 ± 22.63	39.73 ± 45.66
E_2_ (pg/mL)	97.77 ± 116.95	109.96 ± 116.26	66.24 ± 87.95
TSH (μIU/mL)	1.42 ± 0.65	1.79 ± 1.28	2.16 ± 1.34
Abnormally high TSH levels (n, %)	0 (0)	2 (4.3)	4 (8.5)
Free T_4_ (μg/dL)	1.08 ± 0.17	1.07 ± 0.15	1.08 ± 0.17
AMH (ng/mL)	0.32 ± 0.54	0.31 ± 0.40	0.43 ± 0.81
TRACP-5b (mU/dL)	251.95 ± 108.39	248.40 ± 119.49	236.21 ± 126.64
BMD (g/cm^2^)	1.11 ± 0.19	1.17 ± 0.14	1.15 ± 0.13
BMD change (g/cm^2^)	0.02 ± 0.02	0.01 ± 0.00	0.03 ± 0.02
BMD rate of change (%/year)	2.07 ± 2.24	-0.64 ± 0.50	-3.03 ± 1.12
Postmenopausal (n, %)	23 (48.94)	15 (31.91)	15 (31.91)
Age at menopause (years)	48.04 ± 2.10	46.8 ± 4.07	45.13 ± 3.76

Data are presented as means ± SD or number of cases (%)

Abnormally high TSH levels: ≥ 4.23 μIU/mL

AMH, anti-Müllerian hormone; BMD, bone mineral density; E_2_, oestradiol; FSH, follicle-stimulating hormone; T_4_, thyroxine; TSH, thyroid-stimulating hormone; TRACP-5b, tartrate-resistant acid phosphatase-5b

## Discussion

The present study’s results indicated that high FSH and low AMH levels were associated with decreased BMD, suggesting that decreased ovarian function was associated with decreased BMD. Further, a greater rate of decrease in BMD was associated with a higher age at menopause. We showed that TSH was associated with the rate of decrease in BMD and that high TSH level constituted a risk factor for future BMD loss.

### Ovarian function and bone mineral density in endometriosis patients

We found that high FSH and low AMH levels were associated with BMD loss in perimenopausal women with endometriosis and demonstrated that a decrease in ovarian function was related to a decrease in BMD. During menopause, FSH secretion from the pituitary gland increases with decreased oestrogen secretion, and high FSH levels persist after menopause [25, 26]. Previous reports have shown an association between elevated FSH levels and decreased BMD in premenopausal women rather than in perimenopausal women [27]. Meanwhile, an association between high FSH levels and decreased BMD was also observed in perimenopausal women [28]. It has also been reported that FSH is involved in the pathophysiology of postmenopausal osteoporosis [29]. The results of the present study supported the previously demonstrated association between elevated FSH levels and decreased BMD. In addition, several previous studies, including meta-analyses, have reported low AMH levels in patients with endometriosis [30–32]. Previous studies investigating the association between AMH and decreased BMD due to primary ovarian insufficiency in premenopausal women found a positive correlation between BMD and AMH, even after removing the influence of age [33]. The results of the present study showed that women with endometriosis had lower AMH levels than women without endometriosis, which is a new finding demonstrating the relationship between ovarian reserve and BMD.

### Relationship between age at menopause and bone mineral density

In the present study, the age at menopause tended to be higher with a greater rate of decrease in BMD. In recent years, meta-analyses have shown that early-onset menopause increases the risk of fractures [34]. The duration of time after menopause and BMI are important factors determining the risk of osteoporosis [35]. Changes in BMD in perimenopausal women have been reported to include a period of rapid bone loss [36, 37]. The present study investigated the relationship between the rate of change in BMD and age at menopause; it is possible that we evaluated the difference between the rapid and slow periods of BMD change.

### Relationship between TSH and bone mineral density

We demonstrated that high TSH levels increased the risk of subsequent BMD loss in perimenopausal patients with endometriosis. Regarding the association between thyroid hormone and BMD, high thyroid hormone levels and TSH suppression therapy have been suggested as risk factors for high turnover osteoporosis [38]. In addition, it is established that in overt hypothyroidism, bone turnover is reduced due to decreased bone resorption and osteoblast function. However, the relationship between hypothyroidism and BMD in adults remains unclear [39]. With regard to the relationship between endometriosis and thyroid diseases, a study in the United States that investigated whether patients with endometriosis experienced more autoimmune disorders and pain showed that hypothyroidism was significantly more common but there was no difference in hyperthyroidism [40]. A meta-analysis investigating the association between endometriosis and autoimmune diseases found no significant association with autoimmune thyroid diseases [41]. Meanwhile, in endometriosis, an association with thyroid autoimmunity has been reported in vitro, suggesting that thyroid hormone and TSH receptors may be involved in ovarian function regulation [42]. The present study demonstrated the relationship between TSH and changes in BMD in patients with endometriosis. This is considered meaningful as a new finding suggesting the involvement of TSH in the pathophysiology of endometriosis and bone metabolism.

### Strengths and limitations

The strength of the present study is that it assessed changes in BMD over time, ovarian function, and thyroid hormone levels in perimenopausal patients with endometriosis in a prospective cohort study. A report on the accuracy of the bone densitometry instruments used in this study showed that the coefficient of variation was 1.36% in a population with a lumbar spine (L2-L4) BMD of 1.328 ± 0.175 g/cm^2^ (mean ± SD) [43]. Since this population had a BMI of 49.6 kg/m^2^ and a BMD higher than the mean of the subjects in the present study, the coefficient of variation of BMD in the present study was much smaller and is assumed to have little impact on the interpretation of the rate of BMD change.

However, we recognise that there are several limitations. First, we did not consider the effect of menstrual cycles on FSH levels. Although this is not an issue for participants undergoing hormone therapy or after menopause, measurements for other participants should have taken the menstrual cycle into consideration. The second limitation is related to AMH measurements. As the present study measured AMH using the conventional testing method, some cases were below the limit of measurement. These cases were regarded as being at the lower limit value, but this could have led to an overestimation of the ovarian function. In recent years, high-sensitivity AMH testing has been applied in clinical practice. Going forward, the use of high-sensitivity AMH testing is expected to enable more rigorous ovarian reserve assessment, and we would like to conduct research that incorporates such high-sensitivity AMH testing. Third, the study did not address the perimenopausal changes in BMD. As the present study involved a small number of cases and had many deviating cases, the changes over time could only be observed once. Therefore, it was not ascertained whether the change in BMD observed was during a period of rapid BMD decrease in each participant. We believe that further studies with larger cohorts, longer follow-up periods, and consideration of the BMD reduction phase of individuals are required.

## Conclusions

Our study demonstrated that high FSH or low AMH levels were associated with decreased BMD in perimenopausal patients with endometriosis. Because high TSH levels also increase the risk of subsequent BMD loss, measurements of ovarian reserve and TSH may be useful in estimating BMD loss in perimenopausal women with endometriosis. These findings could assist in disease management in women with endometriosis. It is necessary to conduct further studies on the relationship between ovarian reserve and BMD using high-sensitivity AMH testing.

## Data Availability

The datasets used and/or analysed during the current study are available from the corresponding author on reasonable request.

## Abbreviations

BMD: Bone mineral density
FSH: Follicle-stimulating hormone
LH: Luteinising hormone
AMH: Anti-Müllerian hormone
BMI: Body mass index
T_4_: Thyroxine
TSH: Thyroid-stimulating hormone
DXA: Dual-energy X-ray absorptiometry
CI: Confidence interval
